# High-Temperature Stable Amorphous Al_2–*x*_(ZrY)_*x*_O_3_ Thin Film Insulators: An Alternative to Crystalline Alumina

**DOI:** 10.1021/acsami.6c03090

**Published:** 2026-03-31

**Authors:** Norma Salvadores Farran, Florentine Scholz, Tobias Martin Huber, Tomasz Wojcik, Astrid Gies, Jürgen Ramm, Klaus Böbel, Szilard Kolozsvári, Peter Polcik, Jakob Rath, Jürgen Fleig, Helmut Riedl

**Affiliations:** † Christian Doppler Laboratory for Surface Engineering of High-Performance Components, 27259 TU Wien, Vienna 1060, Austria; ‡ Analytical Instrumentation Center, 27259 TU Wien, Vienna 1060, Austria; § Institute of Chemical Technologies and Analytics, 27259 TU Wien, Vienna 1060, Austria; ∥ Oerlikon Balzers, 253785 Oerlikon Surface Solutions AG, Balzers 9496, Liechtenstein; ⊥ Plansee Composite Materials GmbH, Lechbruck am See, Bavaria 86983, Germany; # Institute of Materials Science and Technology, 27259 TU Wien, Vienna 1060, Austria

**Keywords:** amorphous insulators, thin films, doped Al_2_O_3_, thermal stability, HiPIMS, impedance spectroscopy

## Abstract

In high-temperature settings, amorphous alumina thin film insulators offer distinct advantages over crystalline materials, especially including a reduction in pinholes and leakage currents. Nonetheless, the phase stability of amorphous structures is a significant factor in determining their performance as insulators over a broad temperature range. With increasing temperatures, amorphous alumina undergoes a series of crystallization processes, resulting in irreversible alterations such as cracking and delamination due to volume changes between the different Al_2_O_3_ polymorphs. In this study, amorphous alumina doped with yttrium and zirconium has been deposited via physical vapor deposition, demonstrating the stability of the amorphous structure up to 1200 °C in ambient air and vacuum. Above 1000 °C, the amorphous films transform into a two-phase system consisting of tetragonal yttria-stabilized zirconia (YSZ) embedded in an amorphous alumina matrix. Compared to undoped alumina, films alloyed with 4.3, 12.2, and 20.7 at. % ZrY exhibited enhanced thermal stability. Samples alloyed with 4–12 at. % ZrY additionally showed stable and reversible resistivity behavior under thermal cycling. Notably, films containing only 4.3 at. % ZrY achieved uncharted resistivity values of (3.33 ± 0.06) × 10^5^ and (2.83 ± 0.07) × 10^5^ Ω·m at 750 and 850 °C, respectively, representing a substantial enhancement relative to undoped alumina, which exhibited a value of (8.33 ± 0.04) × 10^4^ Ω·m at 750 °C.

## Graphical abstract

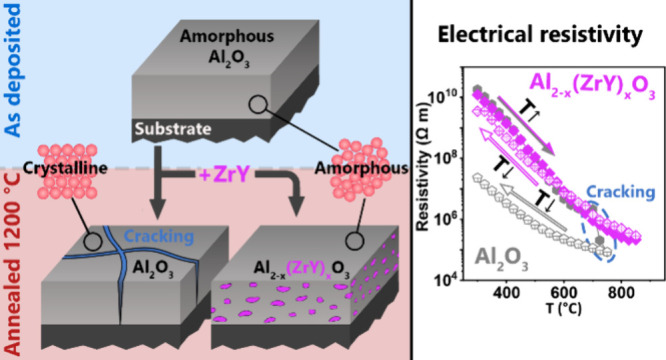

## Introduction

Alumina (Al_2_O_3_) thin films are widely used in advanced technologies due to their excellent thermal stability, corrosion resistance, and high electrical resistivity.^1–3^ In particular, amorphous alumina has recently attracted significant interest as a gate dielectric material for next-generation transistors, motivated by its higher dielectric constant compared to SiO_2_.^4–6^ This enhanced permittivity enables the use of thicker dielectric layers while maintaining high capacitance, thereby reducing leakage currents and supporting continued device scaling.^7^

The amorphous structure of Al_2_O_3_ plays a critical role in its functional performance. The absence of grain boundaries suppresses pinhole formation and leakage pathways commonly observed in polycrystalline films, leading to improved insulating behavior despite a reduction in band gap energy.^8,9^ Structural disorder has also been associated with enhanced thermal transport, further broadening the applicability of amorphous alumina in demanding environments.^10^

Beyond microelectronic applications, amorphous alumina thin films have emerged as promising materials for diverse uses such as transmission electron microscopy (TEM) grids, where their exceptional resistance to oxidation at elevated temperatures is highly advantageous.^11^ Together, these attributes position amorphous Al_2_O_3_ as a versatile dielectric material with relevance across nanoelectronics and high-temperature applications.

However, the amorphous form of Al_2_O_3_ is not the thermodynamically most stable one.^12^ As the temperature is increased, amorphous alumina undergoes different phase transitions to metastable polymorphs and finally to its thermodynamically stable phase, α-Al_2_O_3_ (corundum). This phase transition from κ- to α-Al_2_O_3_ is accompanied by a rapid volume shrinkage that is considered the primary cause of Al_2_O_3_ cracking at high temperatures.^13^

The growth of alumina thin films via Physical Vapor Deposition (PVD) at substrate temperatures below 350 °C typically results in the formation of amorphous films.^14,15^ The crystallization of alumina has been observed to occur at temperatures above 350 °C, supported by high ion fluxes of the growing species^16,17^ promoting diffusion process during film growth and hence crystallization. Schneider et al.^16^ conducted a study on the growth of κ- and θ-Al_2_O_3_ at temperatures ranging from 370 to 430 °C, utilizing the process of so-called reactive ionised magnetron sputtering with the usage of an inductively coupled RF plasma between the cathode and the substrates. While α-Al_2_O_3_ requires high synthesis temperatures, PVD has achieved to grow the thermodynamically stable corundum type structure at reduced temperatures in α-(Cr,Al)_2_O_3_ solid solutions.^18^ Supersaturated solid solutions can form as a result of the rapid cooling rates inherent to PVD.^19^ Nevertheless, Chemical Vapor Deposition (CVD) remains the preferred method for obtaining α-Al_2_O_3_ at temperatures above 1000 °C.^20^

The phase transformation kinetics, especially amorphous to crystalline polymorphs, of Al_2_O_3_ can be modified using alloying elements. As demonstrated by Ragan et al.,^21^ yttrium and chromium dopants in amorphous alumina have been proven to accelerate the phase transition from amorphous to γ-Al_2_O_3_, in comparison to pure alumina. By contrast, the addition of erbium has been observed to retard this process. A detailed study of the thermal stability of amorphous alumina doped with silicon, deposited by magnetron sputtering, has been reported by Bolvardi et al.^22^ It was demonstrated that the presence of silicon increases the on-set temperature for both γ- and α-Al_2_O_3_ by more than 100 °C. The enhancement of thermal stability of γ-Al_2_O_3_ has previously been studied by Nahif et al.,^23^ suggesting that SiO_2_ at the grain boundaries is responsible for the stabilization of the gamma phase. Yttria-stabilized zirconia (YSZ) is also proposed as an alternative alloying route to enhance the thermal stability of amorphous alumina. Liu et al.^24^ demonstrated that the crystallization of alumina could be effectively hindered up to 1000 °C through the process of alloying with 8.6 to 78.3 at. % metallic ZrY. This finding suggests that the formation of t-ZrO_2_ nano crystallites may inhibit the crystallization process of the amorphous Al_2_O_3_ matrix. It has also been observed that the incorporation of nanocrystals into the matrix enhances the toughness and adhesion of the films.^24^ The alteration in the phase transformation kinetics is advantageous for a number of applications where phase transitions and the associated morphology changes should be avoided. To be more precise, the insulation properties of amorphous alumina at elevated temperatures in thin film microelectronics such as sensors or gate insulators is in high demand.^4,12,25^

As demonstrated by Liu et al.,^24^ the composite material of amorphous alumina and YSZ grains exhibits enhanced insulating properties in comparison to pure alumina, attributable to the nanocrystals present within the coating. Furthermore, Bierwagen et al.^26^ demonstrated in their work that ZrAl_*x*_O_*y*_ amorphous thin films do not exhibit leakage currents at low electric fields; this is in contrast to their polycrystalline form.

In this study, the deposition and thermal stability of amorphous alumina thin films insulators doped with yttrium and zirconium is in focus. A range of alloying ratios is explored to identify an optimal balance between maximizing amorphous phase stability and maintaining high electrical insulation at previously unreported elevated temperatures. Alloying concentrations lower than those previously reported in the literature are also examined for this purpose. The depositions were performed using reactive high power impulse magnetron sputtering (HiPIMS). The thermal stability of the amorphous films has been investigated through the utilization of ex-situ X-ray diffraction (XRD). The insulation behavior of the films was analyzed using in situ impedance spectroscopy (IS) up to 850 °C. Transmission electron microscopy (TEM) was used to investigate the morphological properties of annealed samples. Additionally, the stoichiometry and alloying content are assessed through X-ray photoelectron spectroscopy (XPS).

## Results and Discussion

### Reactive Growth of Amorphous Al_2–*x*_(ZrY)_*x*_O_3_-Based Thin Films

To assess the optimal working point for each target material and analyze the stability of the reactive sputter deposition processes, so-called poisoning curves have been performed, see Figure 1. In detail, the four different poisoning curves are summarized, performed through modifying the flow-rate ratio (f_O2_ = O_2_/(Ar + O_2_)), while maintaining the total working pressure constant at around 0.40 ± 0.01 Pa. The observed hysteresis is a result of the compound formation during reactive gas addition (full symbols), and the deaccelerated sputter removal during decreasing f_O2_ flow-rate ratios (open symbols). In relation to the target composition, the formation of the insulating Al_2_O_3_/Al_2–*x*_(ZrY)_*x*_O_3_ compound layer on the target surface also impacts the discharge voltage based on the higher secondary electron yield of Al_2_O_3_ compounds compared to a metallic Al or Al_*x*_(ZrY)_1–*x*_ target surface. This effect alters the shape of the discharge voltage hysteresis and the electrical conductivity of the targets. The surface binding energy (SBE) influences the sputter yield of the target material; when the elemental metal target exhibits a higher sputter yield than the compound, hysteresis behavior occurs (see Figure 1).^27^ In the initial step, oxygen is chemisorbed on the surface of the target without inducing a substantial change in the discharge voltage. At low oxygen partial pressure, the formation of an oxidized target is suppressed by the higher ion bombardment.^27^ This target behavior is reflected by the plateau observed at low reactive gas ratios in Figure 1 and corresponds to the metallic mode of the target, which is characterized by high deposition rates during the growth process.^27,28^ Nevertheless, during a secondary process, oxidation of the target’s surface occurs, resulting in a significant reduction in the discharge voltage (see Figure 1). The voltage decrease is attributed to an increase in the secondary electron emission yield of the oxide compound on the target.^28,29^ This stage occurs when gas absorption by the substrate and chamber walls becomes saturated, leading to an increase in the oxygen partial pressure in the chamber and, consequently, enhanced oxide formation on the target. Such target behavior is referred to as the transition mode. It is typically unstable and difficult to operate due to arcing processes associated with the formation and sputter removal of the compound on the target surface.^27,28^

**Figure 1:**
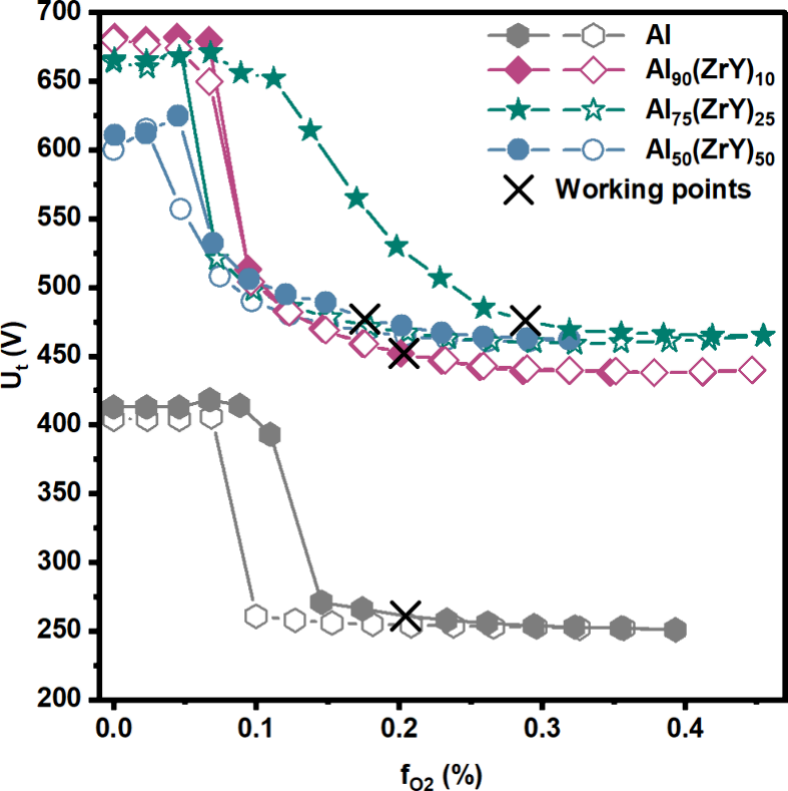
Poisoning behavior of the different Al alloyed targets in a mixed O_2_/Ar gas atmosphere. The diamondsdenote the curve of the Al_90_(ZrY)_10_ target, the stars that of the Al_75_(ZrY)_25_ target, the circles that of the Al_50_(ZrY)_50_ target, and the hexagons that of the reference Al target (in DCMS mode). The filled symbols indicate an increase in the reactive gas ratio, and the empty symbols indicate a decrease. The cross symbols represent the working points of the depositions.

With increasing oxide formation, the sputter yield of the compound on the target decreases, which simultaneously reduces the getter capacity of the substrate. The resulting reduction further increases the oxygen partial pressure, forming a feedback loop and cascade mechanism.^27^ This stage is referred to as the poisoning regime and is characterized by predominant formation of an aluminum oxide compound on the target surface, resulting in altered target conductivity.^27,28^ Consequently, a lower discharge-voltage plateau is observed at higher reactive gas ratios in Figure 1.

It is well documented that the hysteresis behavior can be reduced with the use of HiPIMS.^30^ Anders et al.^31^ attributed this behavior to the dominant role of the ion flux at the target, which is controlled by the deposition parameters, to enhanced self-sputtering of the target, and to the pulsed nature of the process, which prevents full poisoning of the target. This observation is consistent for the AlZrY 90/8.5/1.5 (abbreviated to Al_90_(ZrY)_10_) and AlZrY 50/42.5/7.5 (shortened to Al_50_(ZrY)_50_) targets (nomenclature refers to the atomic fraction). However, for the AlZrY 75/21.25/3.75 (short, Al_75_(ZrY)_25_) target, it can be concluded that the chosen HiPIMS parameters were not the most suitable for reducing the hysteresis in this target. Additionally, for the pure Al target only a stable process in DCMS could be established as the process stability at required high oxygen partial pressure (fo_2_ = 0.26) was very low in HiPIMS mode (at a peak power density of 0.5 kW/cm^2^) and the pure Al target was not capable of such high peak power densities. This behavior also suggests, that alloying Al targets with ZrY enhances the deposition stability, enabling us to run the deposition in HiPIMS mode at peak power densities around 0.5 kW/cm^2^. As demonstrated by Kohlhauser et al. alloying Al targets with Cr, Mo, or W also extends the reactive flow rate ratios and deposition stabilities.^32^

For each target, a working point (denoted by a cross in Figure 1) was selected between the transition and poisoning regime in order to obtain insulating behavior of the Al_2–*x*_(ZrY)_*x*_O_3_ coatings. The growth details are summarized in Table 1. Sample nomenclature is based on the atomic percentage of each element within the coating (quantified by XPS), with the Zr and Y atomic concentrations added up for simplicity – in the general notation Al_2–*x*_(ZrY)_*x*_O_3_ x in at. % is therefore the Zr+Y fraction accounted to the metal sublattice. A clear discrepancy between the target and film compositions is observed. This behavior is consistent with previous reports and can be attributed to several factors inherent to reactive sputtering, including differences in sputtering yields of the target elements,^33^ compound formation on the target racetrack during target poisoning, which alters the sputtering yield compared to the metallic target,^34^ and scattering effects in the plasma.^35^

**Table 1:** Overview of All Depositions, Including the Sample Name, Target Composition, Growth Technique, Reactive Gas Ratio, and Film Composition

thin film	target composition (at. %)	growth technique	reactive gas ratio (%)	film composition (at.%)
Al_0.37_O_0.63_	Al	DCMS	20.5	37.4/62.6 Al/O
Al_0.33_(ZrY)_0.04_O_0.63_	90/8.5/1.5 Al/Zr/Y	HiPIMS	20.1	33.1/3.5/0.8/62.6 Al/Zr/Y/O
Al_0.24_(ZrY)_0.12_O_0.64_	75/21.25/3.75 Al/Zr/Y	HiPIMS	28.8	24.3/10.2/2.0/63.5 Al/Zr/Y/O
Al_0.16_(ZrY)_0.21_O_0.63_	50/42.5/7.5 Al/Zr/Y	HiPIMS	17.6	15.7/16.6/4.1/63.6 Al/Zr/Y/O

### Bonding State

X-ray photoelectron spectroscopy was utilized to determine the composition and the bonding nature of the different elements in the grown Al_2–*x*_(ZrY)_*x*_O_3_ thin films. In order to investigate any change in composition within the surface and the bulk of the coating, the films were sputtered for 20 min. Figure 2 illustrates the bonding energies of Al 2p (Figure 2a), O 1s (Figure 2b), Y 3d (Figure 2c), and Zr 3d (Figure 2d) of the bulk of the coating.

**Figure 2:**
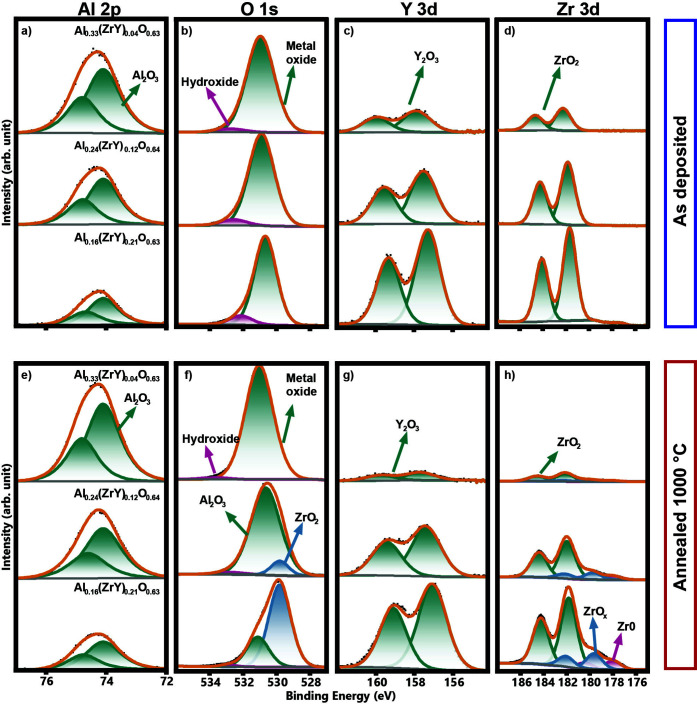
XPS spectra of (a–d) as deposited alloyed samples and (e–h) samples annealed at 1000 °C in vacuum: (top) Al_0.33_(ZrY)_0.04_O_0.63_, (middle) Al_0.24_(ZrY)_0.12_O_0.64_, and (bottom) Al_0.16_(ZrY)_0.21_O_0.63_. (a and e) Al 2p peak and envelope compressing the spin–orbit subpeak pairs. (b and f) O 1s peak with the fitting enveloped and the subpeaks. (c, d, g, and h) Y 3d peak and Zr 3d peak and their envelope, including the spin–orbit subpeak pairs.

Figure 2a depicts the Al 2p peak, where the solid orange line indicates the envelope of the contribution from all subpeaks. Only one chemical state was observed at 74.1 eV binding energy which can be attributed to a Al–O bond.^36^ The peak is split into two distinct peaks pairs (2p_3*/*2_ and 2p_1*/*2_) with an energy difference of 0.6 eV. As the alloying content is increased, the intensity of the Al 2p decreases, indicating a reduced relative concentration of aluminum. No chemical shift is observed within the spectrometer resolution of 0.2 eV.

Figure 2b shows the O 1s peak, which is the sum of two contributions from subpeaks. The peak at the lowest binding energies (530.8 ± 0.1 eV) can be attributed to lattice oxygen in metal oxides^36^ with no possibility to distinguish between the O–Al or O–Zr bonding due to the overlap of the subpeaks. The second subpeak is identified as hydroxyl groups (532.6 ± 0.1 eV). Again, no chemical shift is observed within the spectrometer resolution of 0.2 eV.

Figure 2c and d represent the Y 3d and Zr 3d peaks, which respectively correspond to sup-peak splitting of 3d_5/2_ and 3d_3/2_. Both elements exhibit a bond only to oxygen, which can be corroborated by the position of the peaks (Y–O bond: 157.6 ± 0.3 eV and Zr–O bond: 182.1 ± 0.3 eV, in average). This is in agreement with Y^3+^ and Zr^4+^ oxidizing states,^36^ thereby indicating the presence of Y_2_O_3_ and ZrO_2_ components. A clear increase in intensity is observed as the alloying content was increased in the films. Nevertheless, with increasing ZrY content, Zr 3d and Y 3d core levels shift toward lower binding energy (a difference of 0.6 eV), indicating an increased electron density around the cations. In contrast, the O 1s peak position remains nearly unchanged due to the coexistence of multiple oxygen coordination environments with similar binding energies.

Moreover, no additional shift (within the resolution of the spectrometer) regarding the reference sample is observed (Figure S1, Supporting Information). This indicates a similar chemical environment in all Al_2–*x*_(ZrY)_*x*_O_3_ films with amorphous nature. The estimated compositions of the films are summarized in Table 1.

Further investigations have been performed on the samples annealed at 1000 °C in vacuum, with the aim to evaluate any potential change in the bonding state (Figure 2e–h). A significant difference can be observed in the oxygen peak. For the Al_0.16_(ZrY)_0.21_O_0.63_, the metal oxide peak can be resolved, and it can be seen that the major component is oxygen bonded to zirconium. This indicates a higher contribution of ZrO_2_ bonding, which is in agreement with the Zr rich phases in the annealed samples (see further section). In addition, the Zr 3d peak demonstrates the presence of additional oxidation states with a more reduced nature than ZrO_2_, which was the only peak present on as deposited state. The emergence of reduced oxide peaks can be attributed to the presence of oxygen vacancies. During annealing in vacuum, oxygen atoms are known to desorb from the crystal lattice, thereby leading to a partial reduction of Zr^4+^. However, no significant energy shift is observed compared to the as deposited state.

### Thermal Stability

The thermal stability of all grown films was analyzed by thermal treatments accompanied by X-ray diffraction. The diffractograms were recorded ex-situ after annealing the samples in ambient air for 100 min at temperatures of 600, 800, 1000, and 1200 °C. The results are summarized in Figure 3. At the bottom of the figure, an uncoated sapphire substrate diffractogram is represented as a reference for the substrate peaks. As highlighted in Figure 3a, the reference Al_2_O_3_ film crystallizes at 800 °C to α-Al_2_O_3_, exhibiting a peak at 68.2° corresponding to the (300) reflection (ICDD: 00–010–0173). It is notable that no phase transition to γ-alumina is observed at these temperature steps and annealing times. In contrast, for all Al_2–*x*_(ZrY)_*x*_O_3_ thin films, no crystalline Al_2_O_3_ peak is indexable, suggesting that the Al_2–*x*_(ZrY)_*x*_O_3_ films maintain an amorphous state up to a temperature of 1200 °C. Due to ZrY alloying the thermal energy required to promote the nucleation of Al_2_O_3_ is obviously not high enough at 1200 °C. However, the crystallization of t-YSZ (ICDD: 00–060–0501) is recognizable with the main intensity peak at 30.1° and 35.0° corresponding to (101) and (110) planes of the tetragonal phase. The crystallization of t-YSZ is observed at lower temperatures for the film containing the highest amount of ZrY, Al_0.16_(ZrY)_0.21_O_0.63_ with 16.6 and 4.1 at. % of Zr and Y, respectively. In their previous work, Liu et al.^24^ have already described the delay of alumina crystallization by YSZ nanocrystallites up to 1000 °C. Interestingly, in this study it has been observed that the phase stability can be extended by a minimum of at least to 200 °C more, while employing a significantly lower alloying concentration of around 4 at. % ZrY in total. Diffractograms using a three-omega tilt have been also recorded to avoid or reduce the signal of the substrate peaks, obtaining the same trends (see Figure S2 in the Supporting Information).

**Figure 3:**
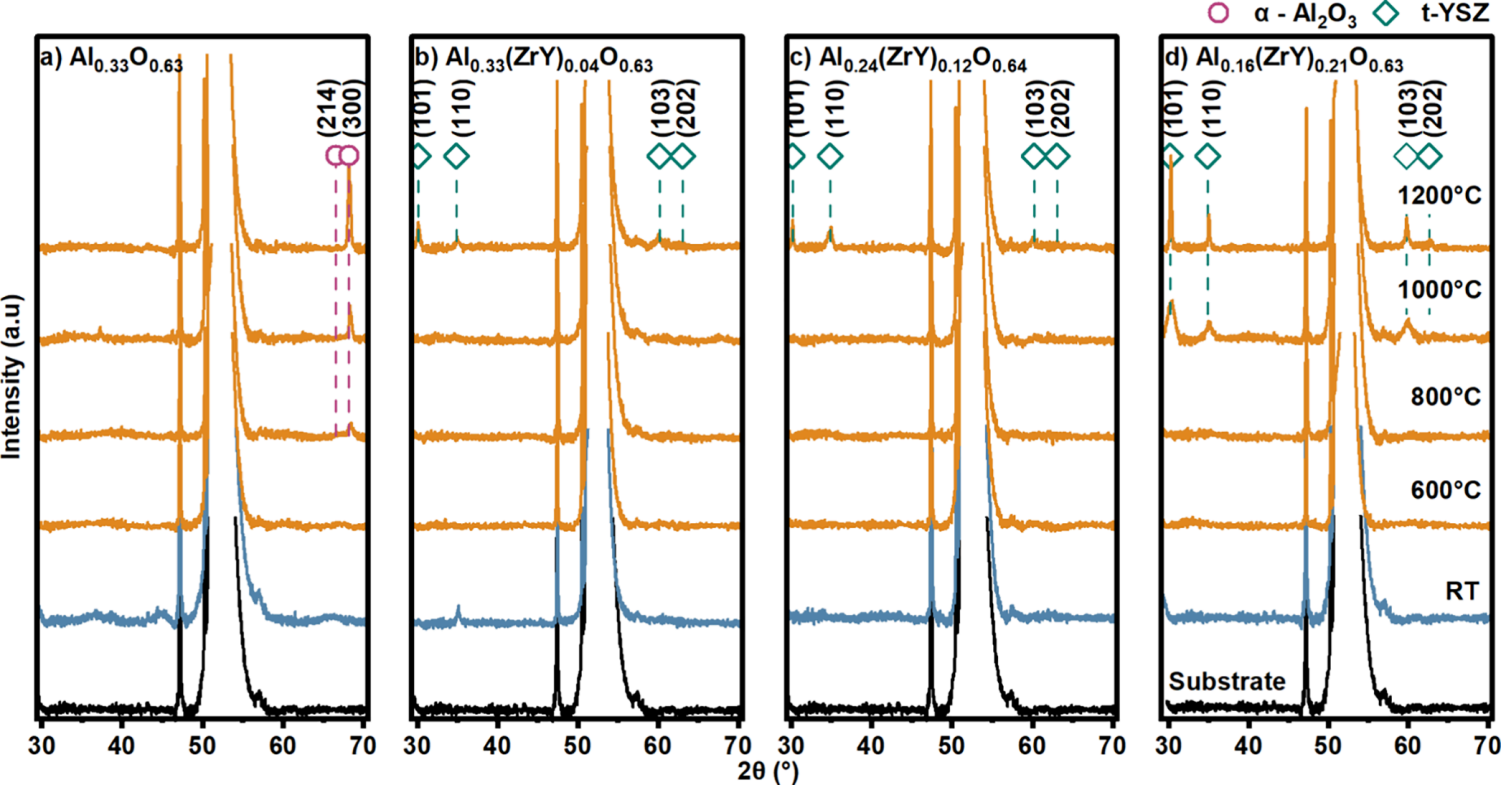
XRD diffractogram of as deposited and annealed samples in ambient air from 600 to 1200 °C in 200 °C temperature steps. Substrate diffractograms represented on the bottom as a reference: (a) Al_0.37_O_0.63_, (b) Al_0.33_(ZrY)_0.04_O_0.63_, (c) Al_0.24_(ZrY)_0.12_O_0.64_, and (d) Al_0.16_(ZrY)_0.21_O_0.63_.

To cross check the observed behavior also annealing treatments under vacuum conditions (1 × 10^–6^ mbar) have been performed (see Figure S3, Supporting Information). Here, the thermal stability of the amorphous materials shows minor differences. In line with to the results in Figure 3, Al_2–*x*_(ZrY)_*x*_O_3_ films also obtain no crystalline Al_2_O_3_ peaks. However, for the unalloyed Al_2_O_3_ reference sample, the crystallization also shifted to 1200 °C, which is 400 °C more compared to annealing treatments in ambient air. In addition, annealing at 1400 °C even confirmed these results with no other peaks identified (see Figure S3). This suggests that more oxygen is needed for crystallization, indicating that the annealing atmosphere influences the nucleation and growth rates of crystalline phases in nonstoichiometric compounds (Al_0.37_O_0.63_) during annealing.

In ambient air, the oxygen partial pressure is drastically higher than in the vacuum furnace, thereby facilitating oxygen diffusion and supporting the process of crystallization. A comparison of the two annealing methods revealed the presence of identical t-YSZ peaks, suggesting comparable crystallization processes.

A detailed microstructural investigation was performed using cross sectional transmission electron microscopy (TEM) after annealing at 1200 °C for 100 min in vacuum atmosphere (1 × 10^–6^ mbar). The results obtained for the Al_0.33_(ZrY)_0.04_O_0.63_ sample are summarized in Figure 4. In Figure 4a, a bright field image is presented, which demonstrates the presence of grains/precipitates distributed within the alumina matrix. These grains correspond to crystalline yttria-stabilized zirconia (YSZ). Figure 4c provides a more detailed view of this region. Figure 4b offers an alternative view of the image, showcasing the dark field image and emphasizing the bright spots that indicate crystalline reflections, occupying only a small area of the full coating – see also chemical maps in Figure 4g and h. The analysis further demonstrates that the matrix remains amorphous, as no reflection was observed. In order to verify the amorphous nature of the matrix, a high-resolution TEM was performed on the area indicated in Figure 4c. Figure 4d confirms the amorphous nature of the remaining matrix, as there is no long-range order observed. In addition, the Fourier transform of the image confirms the amorphous nature of the matrix – see Figure 4e. The presence of a two-phase system is indicated by the bright and dark field images, a conclusion that is further substantiated by the scanning TEM (STEM) image and electron energy loss spectroscopy (EELS) elemental maps (Figure 4f-i). These maps demonstrate the complementary behavior of Al and Zr. For the matrix maybe amorphous Al_2_O_3_ rich regions as well as Al_2–*x*_(ZrY)_*x*_O_3_ rich regions can be distinguished. However, Y elemental mapping was added alongside Zr mapping due to its weak signal and low concentration. In the Supplementary, EELS mapping for the other two alloyed samples are also depicted in Figure S4 (Supporting Information), thereby validating the existence of a two-phase system across all compositions.

**Figure 4:**
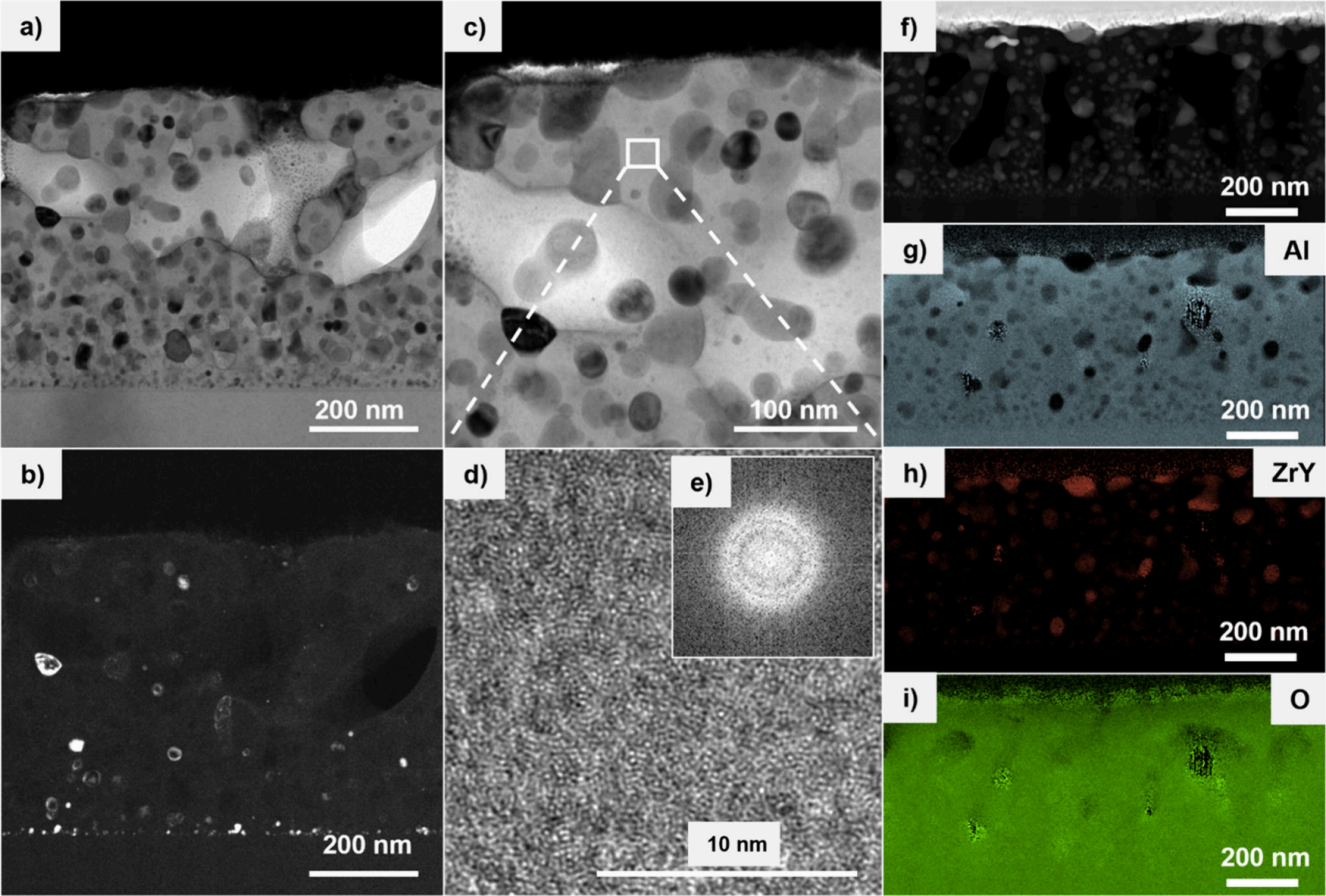
(a) TEM bright-field micrograph of Al_0.33_(ZrY)_0.04_O_0.63_ annealed at 1200 °C, (b) corresponding dark-field image, and (c) magnified view of panel a. (d) High-resolution TEM image of the area marked in panel c. (e) Fast Fourier transform (FFT) of the image in panel d. (f) STEM image of Al_0.33_(ZrY)_0.04_O_0.63_ used for elemental mapping, with corresponding maps of (g) Al, (h) ZrY, and (i) O.

The TEM results are in agreement with the structural analysis by XRD, which also suggest that the t-YSZ phase has undergone a process of crystallization but not the amorphous Al_2–*x*_(ZrY)_*x*_O_3_ matrix. Furthermore, ToF-SIMS profiles were used to confirm the homogeneously distributed alloying content along the depth of the as deposited film (Figure S5, Supporting Information). However, after annealing at 1200 °C there is an Al depletion at the surface accompanied by a slight increase of Zr and Y at the surface near region due to diffusion.

Moreover, the ToF-SIMS elemental mapping (see Figure S6 in the Supporting Information) reveals the diffusion and segregation of Zr and Y post annealing, resulting in size distributions of approximately 10 μm. These elements are randomly distributed within the alumina matrix.

### Electrical Properties

In situ impedance spectroscopy was used to assess the insulating behavior of the Al_2–*x*_(ZrY)*_x_* O_3_ films. The temperature dependence of electrical resistivity was investigated from 300 to 850 °C. Figure 5a illustrates the Nyquist plot for all samples at 650 °C during the heating procedure. The fitting was performed with *ZView* using an equivalent circuit of two blocks of a resistor in parallel with a constant phase element (CPE). The primary block was correlated with the resistivity of the bulk of the coating, given its capacitance value, see below, while the subsequent block was incorporated to fit the low frequency regime of the spectra. This resistance is likely associated with a charge transfer step at the interface between the film and the Pt electrode due to the high-capacitance values. Therefore, only the high frequency semicircle is attributed to the alumina-based films. The calculation of the film resistivity (ρ) was performed on the basis of the resistance obtained through the fitting and the geometry factors of the film.

**Figure 5:**
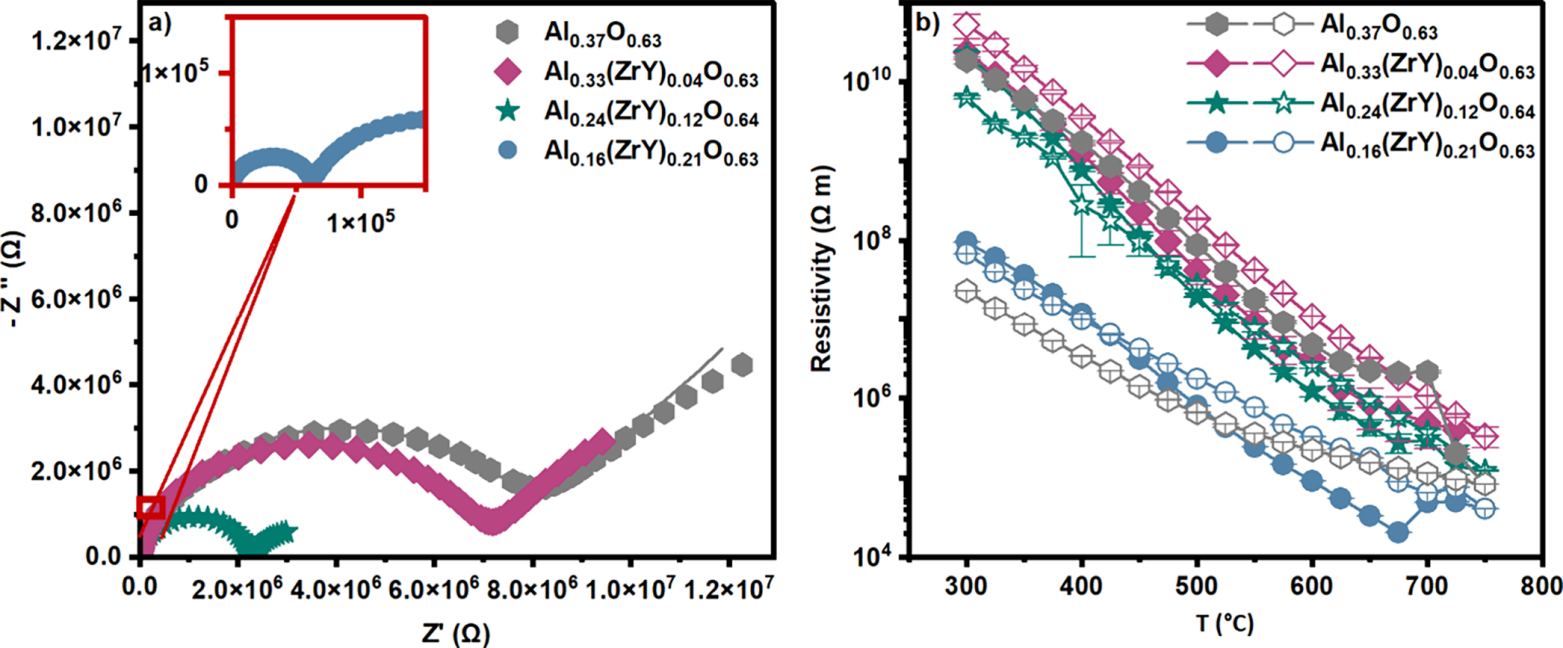
(a) Experimental Nyquist plots for all samples measured at 650 °C (symbols), with equivalent-circuit fits shown as solid lines. (b) Temperature dependence of the electrical resistivity for all deposited films. Filled symbols correspond to data acquired during heating, while empty symbols represent measurements obtained during cooling.

Figure 5b depicts the electrical resistivity of all films as a function of the temperature – with full symbols are resistivity values measured when heating up and the empty symbols are the ones measured when cooling down in the temperature cycle. It is evident that all samples exhibit a decline in the electrical resistivity with an increase in temperature, as expected for ceramic materials. However, Al_0.33_(ZrY)_0.04_O_0.63_ and Al_0.24_(ZrY)_0.12_O_0.64_ exhibit an almost reversible behavior when cooling down, thereby indicating also a high thermal stability of the material. Al_0.16_(ZrY)_0.21_O_0.63_ exhibit a pronounced increase in resistivity at 675 °C, which is attributed to the relaxation of defects in the film. Upon cooling, the Al_0.16_(ZrY)_0.21_O_0.63_ film exhibits enhanced insulating properties. The presence of higher concentrations of Zr and Y in the coating strongly affects the electrical resistivity of the film, which is approximately 2 orders of magnitude less compared to the less alloyed films – see the blue circles for Al_0.16_(ZrY)_0.21_O_0.63_. This phenomenon can be attributed to the elevated ionic conductivity exhibited by YSZ in comparison to alumina.^37^

In contrast, the pure amorphous Al_0.37_O_0.63_ film features a distinctly different behavior. At 700 °C, a sharp decrease in the resistivity values take place, after which the resistivity remains at a substantially lower level compared to the initial recorded values during the heating cycle. This sudden decrease can be attributed to a phase transition of alumina at this temperature (see Figure 3a), which may lead to crack formation due to mismatches in thermal expansion coefficients or to morphological changes in the films (see Figure S7 for visual evidence of these alterations). These alterations can facilitate electron mobility, thereby increasing the conductivity of the film. Resistance values recorded during the cooling stage of the temperature cycle are summarized in Table S1 (see Supporting Information).

As Al_0.33_(ZrY)_0.04_O_0.63_ is the sample which presents improved insulating properties and reversible behavior, an additional temperature cycle up to 850 °C (Figure 6 – see cycle 2 green symbols) has been measured to study its behavior at higher temperatures where pure alumina already crystallizes (Figure 3a). The sample exhibits an almost reversible behavior reaching values of (2.8 ± 0.7) × 10^5^ Ω·m at 850 °C representing uncharted terrain so far. Moreover, comparing this result with different crystalline/amorphous aluminum oxide literature values,^38–41^ the outstanding performance of amorphous ZrY dopped alumina is highlighted, as it presents similar resistivity values than α-Al_2_O_3_ at these high temperatures and even further on.^41^

**Figure 6:**
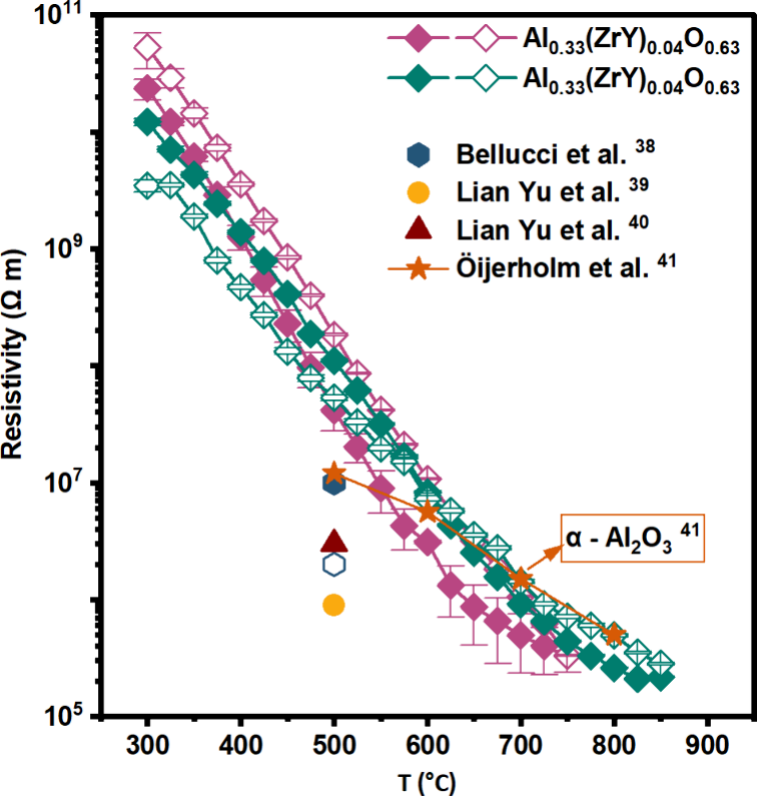
Electrical resistivity of Al_0.33_(ZrY)_0.04_O_0.63_ measured during the first thermal cycle (300 to 750 °C, pink diamonds) and the second thermal cycle (300 to 850 °C, green diamonds). Filled symbols represent data acquired during heating, while empty symbols correspond to cooling. Literature data for comparison are included: blue octahedra (filled, amorphous; empty, crystalline Al_2_O_3_) from ref^38^, yellow circles (polycrystalline Al_2_O_3_) from ref^39^, red triangles (polycrystalline Al_2_O_3_) from ref^40^, and orange stars (crystalline Al_2_O_3_) from ref^41^.

The activation energy calculation in the high-temperature range (450 to 750 °C) was derived from Arrhenius plots of the conductivity data. In Table 2, the data for each sample and each stage of the heating and cooling cycle is presented. The value obtained in the heating cycle of Al_2_O_3_ is consistent with the literature for this temperature range, since values of 1.34 eV have been reported.^24^ However, following the sharp decrease in conductivity at around 700 °C, the activation energy is reduced to 0.66 eV. It is evident that Al_0.33_(ZrY)_0.04_O_0.63_ and Al_0.24_(ZrY)_0.12_O_0.64_ exhibit comparable values to those documented in the existing literature on Al_2_O_3_ and also demonstrates a reversible response to temperature cycles. Nevertheless, the sample containing a higher concentration of YSZ (Al_0.16_(ZrY)_0.21_O_0.63_), which also exhibits reduced resistivity, demonstrates a comparatively low activation energy. These results again highlight the importance of ZrY alloying in improving the thermal stability of amorphous alumina, as well as its influence on the insulating properties of the films at high temperatures.

**Table 2:** Activation Energies Extracted from Resistivity Data Recorded during the Heating and Cooling Stages of the Thermal Cycles

sample	*E*_a_ during heating (eV)	*E*_a_ during cooling (eV)
Al_0.37_O_0.63_	1.370 ± 0.004	0.660 ± 0.002
Al_0.33_(ZrY)_0.04_O_0.63_	1.390 ± 0.006	1.530 ± 0.002
Al_0.24_(ZrY)_0.12_O_0.64_	1.410 ± 0.004	1.330 ± 0.006
Al_0.16_(ZrY)_0.21_O_0.63_	1.238 ± 0.001	0.940 ± 0.003

To evaluate whether the conductivity mode of the doped samples was electronic or ionic, we modified the voltage amplitude of the AC signal from 10 to 300 mV and, furthermore, applied an additional DC voltage of up to ±300 mV. When only changing the AC amplitude the spectra remained unchanged, indicating that the sample stayed within a time-independent linear regime throughout the measurements. This supports our initial assumption that the high frequency arc is caused by a bulk process rather than an interfacial phenomenon.

When applying a DC bias during impedance measurements, however, a shift in the spectrum is observed. In particular, the size of the high frequency semicircle changes with bias voltage. These changes are also observed when performing the measurement at different AC amplitudes. Such a modification of the bulk semicircle or bulk resistance is expected when (some) ionic species are mobile in the bulk but electrically blocked at the interfaces to the electrodes. Then, a DC bias leads to a redistribution of the internal ion or ionic defect concentration and thus also to a modification of the local conductivities. This is seen as a change of the (integral) bulk resistance. Hence, these results are in agreement with the assumption that the primary type of conductivity is ionic, rather than electronic.

The determination of the dielectric constant at 650 °C of each material can be made using the following eq 1, derived from the parameters obtained on the fitting of an equivalent circuit of the Nyquist plots:

1 $$\varepsilon =\frac{C_{edd}d}{\epsilon_{0}A}=\frac{{\left(R^{1-n}Q\right)}^{1/n}d}{\varepsilon_{0}A}$$

where C_eff_ is the effective capacitance of the film, R is the resistance of the film, d the film thickness, A the electrode area, Q is the CPE prefactor and n the CPE exponent.^42,43^

The values of the reference alumina depicted in Table 3 is in agreement with the reported literature data, where values around 9 ^44,45^ are reported.^44,45^ As demonstrated in Table 3, an increase in the alloying content of Zr and Y can be associated with an increase in dielectric constants. This increase is anticipated, as previous studies have reported, that alumina obtains a dielectric constant of approximately 9 and amorphous YSZ of around 26.^46^ It is assumed that the effective dielectric constant can be calculated using the linear mixing rule (*ε* = *ε*_*Al*_2_ *O*_3__ *f*_*Al*_2_ *O*_3__ + *ε*_*YSZ*_ *f*_*YSZ*_, where f_*x*_ is the volume fraction of each phase calculated from the atomic composition and assuming ideal stoichiometry and bulk molar volumes). Results are represented in the third column of Table 3. This enables verification of the increase in dielectric constant with increasing alloy content.

**Table 3:** Experimental Dielectric Constants Obtained through a Fit of Nyquist Plots and Calculated Dielectric Constants from the Linear Mixing Rule^a^

sample	dielectric constant	dielectric constant from linear mixing
Al_2_O_3_ (literature)	9.0,^44^ 9.63 ^45^	
YSZ (literature)	26.4 ^46^	
Al_0.37_O_0.63_	9.7 ± 2.0	–
Al_0.33_(ZrY)_0.04_O_0.63_	12.3 ± 1.6	11.72
Al_0.24_(ZrY)_0.12_O_0.64_	13.3 ± 0.1	16.48
Al_0.16_(ZrY)_0.21_O_0.63_	16.4 ± 0.2	20.39

a Literature values for Al_2_O_3_ and YSZ are included for comparison.

## Conclusions

This study evaluates the thermal stability and electrical conductivity of amorphous alumina doped with different concentrations of Zr and Y. The results demonstrate that Al_2–*x*_(ZrY)_*x*_O_3_ films remain in amorphous form up to 1200 °C, with no clear indication of crystalline alumina via XRD and TEM analysis. Compared to the reference unalloyed alumina the crystallization of any Al_2_O_3_ polymorphs can be retarded through ZrY alloying by at least 400 °C in ambient air. Interestingly, the precipitation of YSZ crystallites at temperatures near 1200 °C has been shown to inhibit the crystallization of the amorphous Al_2_O_3_ matrix. In addition, Y and Zr strongly stabilizes the HIPIMS growth process as they allow depositions at higher oxygen partial (up to f_O2_ = 0.3) pressures at simultaneously higher peak power densities compared to DCMS processes. The XPS results indicate that Al and Zr do not form a bond with each other; instead, each element is individually bonded to oxygen. This finding supports the formation of a two-phase system at elevated temperatures, in which t-YSZ crystallizes within an amorphous Al_2–_ *_x_* (ZrY)*_x_* O_3_ matrix, accompanied by the emergence of different grain sizes, as revealed by high-resolution TEM analysis. As Zr and Y strongly impact the thermal stability of amorphous Al_2_O_3_-based films, the electrical properties of Al_2–*x*_(ZrY)_*x*_O_3_ are also altered when tested at elevated temperatures. Although an increase in metallic Zr and Y in the coatings would be associated with a decrease in electrical resistivity, the samples do not exhibit a significant reduction in electrical resistivity. High-temperature in situ impedance spectroscopy reveals some intriguing results. Unalloyed Al_0.37_O_0.63_ exhibited an apparent phase transition/morphological change at around 700 °C accompanied by crack formation. In sharp contrast, the Al_0.33_(ZrY)_0.04_O_0.63_ and Al_0.24_(ZrY)_0.12_O_0.64_ films exhibit no changes during temperature cycling between 300 and 750 °C. In more detail, the best performing sample containing only 3.5 at. % of Zr and 0.8 at. % Y exhibits an electrical resistivity of (3.33 ± 0.06) × 10^5^ at 750 °C and (2.83 ± 0.07) × 10^5^ Ω·m at 850 °C, obtaining reversible behavior during thermal cycling. These values substantially exceed the resistivity of undoped alumina ((8.33 ± 0.04) × 10^4^ Ω·m at 750 °C) and known literature data for crystalline Al_2_O_3_. Moreover, the reversible insulating behavior observed up to 850 °C, with resistivity values comparable to crystalline alumina, highlights the capability of Al_2–*x*_(ZrY)_*x*_O_3_ to stabilize the amorphous phase while maintaining excellent insulating properties – even small YSZ crystallites form within the matrix. Furthermore, it has been demonstrated that alloying can effectively tune the activation energy and dielectric constant of Al_2–*x*_(ZrY)_*x*_O_3_ thin films from 9.7 to 16.4.

## Experimental Section

### Film Growth

All coatings were deposited in an in-house developed laboratory-scale magnetron sputtering system operated in HiPIMS mode, with the exception of the reference Al_2_O_3_ sample, which was grown in DCMS. The films were grown in a reactive Ar/O_2_ gas atmosphere at a total working pressure of 0.4 Pa. Different 3″ aluminum targets with varying alloying contents (see Table 1), were provided by Plansee Composite Materials GmbH. For all depositions, the substrate temperature was maintained constant at 270 °C and an average power density of about 5.50 W/cm^2^ for both HiPIMS and DC processes. The pulse shape during HiPIMS was set to a duty cycle of 2.5 % at a frequency of 1000 Hz, in order to obtain a peak power density of around 0.5 kW/cm^2^.

All films were deposited on silicon substrates (100-oriented, 20 × 7x0.38 mm^3^), sapphire (101̅1-oriented, 10 × 10 × 0.53 mm^3^) and polished Inconel 718 (10 × 10 × 3 mm^3^). Inconel 718 was utilized as a metallic substrate for electric characterization, whereas silicon and sapphire were employed for morphology and phase stability determination. The distance between the substrate and the target was maintained constant at 70 mm for all depositions. The samples were positioned parallel to the target and rotated at a frequency of 0.25 Hz. Prior to the deposition process, the substrates were cleaned in an ultrasonic bath in acetone and ethanol for 5 min each. Subsequently, the samples were positioned within the deposition chamber and subjected to Argon etching for 10 min, with a working gas pressure set at 4.9 Pa and the bias potential adjusted to 800 V. Prior to each deposition, the targets were subjected to a presputtering process behind a closed shutter. This process was intended to precondition the target and establish more stable deposition conditions.

### Coating Characterization

The crystal structure was investigated on sapphire substrates by X-ray diffraction (XRD) analysis in Bragg–Brentano mode. The measurements were performed using a Panalytical XPert Pro MPD system, which was equipped with a Cu–K_α_ radiation source (wavelength λ = 1.54 Å). All measurements were performed between 10 and 100 ° without rotation.

Transmission electron microscopy (TEM) investigations were conducted using FEI TECNAI F20 TEM system, operating at 200 kV, to obtain a described information regarding the morphology of the coating. Additionally, electron energy-loss spectrometry (EELS) mapping was utilized for the chemical analysis of the annealed samples. The TEM lamellas were produced vias a standard FIB lift-out method.

The electrical resistivity was investigated through the utilization of in situ impedance spectroscopy, employing a high-resolution dielectric analyzer (AIS Alpha, Novocontrol, Germany). For this study, microelectrodes with a thickness of 10 nm Ti and 100 nm Pt, and a diameter of 600 μm, were sputtered following a photolithography process. Platinum probe needles ensured the electrical contact between the microelectrode on top of the coatings and the counter-electrode. The samples were placed in a microcontact test station (Huber Scientific, Plug&Probe Micro) inside a glass tube within a furnace, where all measurements were performed in ambient air. The temperature was measured with a thermocouple. A temperature cycle from 300 to 750 °C was recorded with 25 °C increment. At each temperature step, three impedance spectra were recorded per electrode and two electrodes were measured consequently in order to gain a better understanding of the sample behavior. Moreover, two samples with the same composition were measured for better statistics. The measurements were conducted within a frequency range from 10^6^ Hz to 1 Hz, using an AC amplitude of 100 mV and no bias was applied.

X-ray photoelectron spectrometry (XPS) analysis was conducted on the Versaprobe III to study the bonding nature and elemental composition of the different samples. Monochromatic X-ray source (Al K_α_) with a focusing diameter of less than 10 μm and an argon_2500_ cluster source was utilized for removal of organic contaminants and for profile investigations. The utilization of dual-beam charge neutralization, employing low-energy argon ions and electrons, was necessitated by the insulator nature of the coatings. The data was analyzed using CasaXPS. The baseline of the peaks was determined by Shirley background subtraction model and all subpeaks were fitted with a combination of Gaussian and Lorentzian line shapes. The fitting parameters were obtained from the online database xpsfitting.com. For the binding energy calibration, the Adventitious carbon primary peak on the surface was used and set to 284.8 eV.^47,48^ In the investigation of the bulk of the coating, where no Adventitious carbon was detected, the position of the Al 2p peak on the surface was used as a reference. Finally, the atomic concentration was determined with the help of the atomic sensitivity factors provided by the manufacturer.

Surface imaging and in-depth analyses were performed using a TOF–SIMS 5 instrument (ION-TOF GmbH, Münster, Germany) equipped with a BiMn alloy liquid metal ion gun operated at 25 keV for analysis and a dual-source column operated at 2 keV using O_2_^+^ ions for sputtering. The as-deposited and annealed samples were mounted on a Backmount sample holder, introduced into the main chamber, and analyzed under ultrahigh vacuum conditions (∼6 × 10^–8^ mbar).

For depth profiling/sputtering, a 2 keV O_2_^+^ ion beam (∼635 nA, raster size 300 × 300 μm2) from the dual-source column was employed to monitor the diffusion of Y–Zr cations (89Y^+^ and 90Zr^+^) within the thin films. A Bi^+^ primary ion beam (0.28 pA, raster size 100 × 100 μm2) was used for all samples in noninterlaced CBA mode,^49,50^ applying a sputter cycle of 1 s followed by a 1 s pause with an active flood gun. Data were acquired with a cycle time of 50 μs and a random raster over 512 × 512 pixels. To compensate for surface charging effects, a surface potential of – 88.5 V was applied in addition to an extraction bias of 40 V.

## Supplementary Material



## Notes

XPS Al 2p and O 1s spectra of as deposited samples (Figure S1), XRD diffractogram with three omega tilt (Figure S2), XRD diffractogram of vacuum annealed samples (Figure S3), STEM and EELS elemental mapping of Al_0.24_(ZrY)_0.12_O_0.64_ and Al_0.16_(ZrY)_0.21_O_0.63_ (Figure S4), SIMS depth profile of Al_0.16_(ZrY)_0.21_O_0.63_ (Figure S5), ToF-SIMS elemental mapping of Al_0.16_(ZrY)_0.21_O_0.63_ (Figure S6), light microscope image of the Al_0.37_O_0.63_ sample after thermal cycling (Figure S7), and electrical resistivity values versus temperature (Table S1) (PDF)
