# Supplementary material for: High-Temperature Stable Amorphous Al2–x (ZrY) x O3 Thin Film Insulators: An Alternative to Crystalline Alumina
Source: ACS Appl Mater Interfaces. 2026 Mar 31;18(14):21308–18. doi: 10.1021/acsami.6c03090 (PMC13088040; doi:10.1021/acsami.6c03090)
Supplement: Supplementary file 1 [file am6c03090_si_001.pdf]

Supporting Information

High-temperature stable amorphous $Al_{2-x}(ZrY)_xO_3$ thin film insulators – An alternative to crystalline alumina

Norma Salvadores Farran[1*], Florentine Scholz[2,3], Tobias Martin Huber[3], Tomasz Wojcik[1], Astrid Gies[4], Jürgen Ramm[4], Klaus Böbel[4], Szilard Kolozsvári[5], Peter Polcik[5], Jakob Rath[2,3], Jürgen Fleig[3], and Helmut Riedl[1,6]

1 Christian Doppler Laboratory for Surface Engineering of high-performance Components, TU Wien, Vienna, Vienna, 1060, Austria
2 Analytical Instrumentation Center, TU Wien, Vienna, Vienna, 1060, Austria
3 Institute of Chemical Technologies and Analytics, TU Wien, Vienna, Vienna, 1060, Austria
4 Oerlikon Balzers, Oerlikon Surface Solutions AG, Balzers, 9496, Liechtenstein
5 Plansee Composite Materials GmbH, Lechbruck am See, Bavaria, 86983, Germany
6 Institute of Materials Science and Technology, TU Wien, Vienna, Vienna, 1060, Austria

norma.salvadores@tuwien.ac.at*

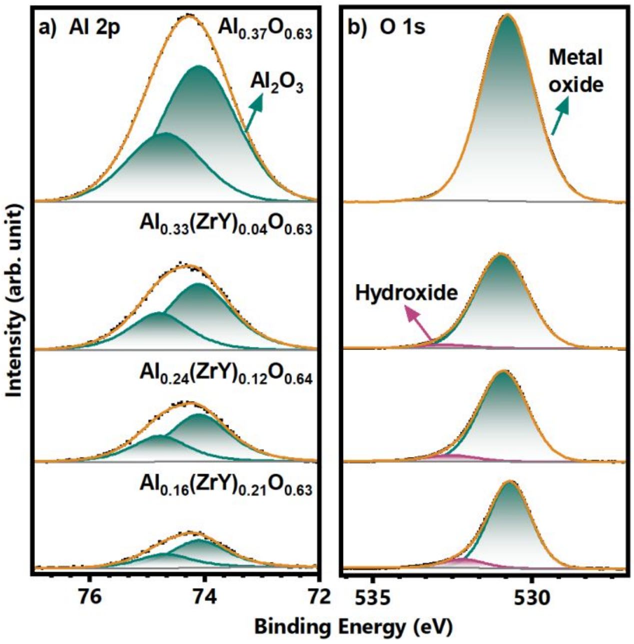

Figure S1: XPS spectra of as deposited alloyed samples. From top to bottom: $Al_{0.37}$ $O_{0.63}$, $Al_{0.33}(ZrY)_{0.04}$, $O_{0.63}$, $Al_{0.16}(ZrY)_{0.21}O_{0.63}$, $Al_{0.24}(ZrY)_{0.12}O_{0.64}$. (a) Al 2p peak and envelope compressing the spin-orbit subpeak pairs. (b) O 1s peak with the fitting enveloped and the sub-peaks.

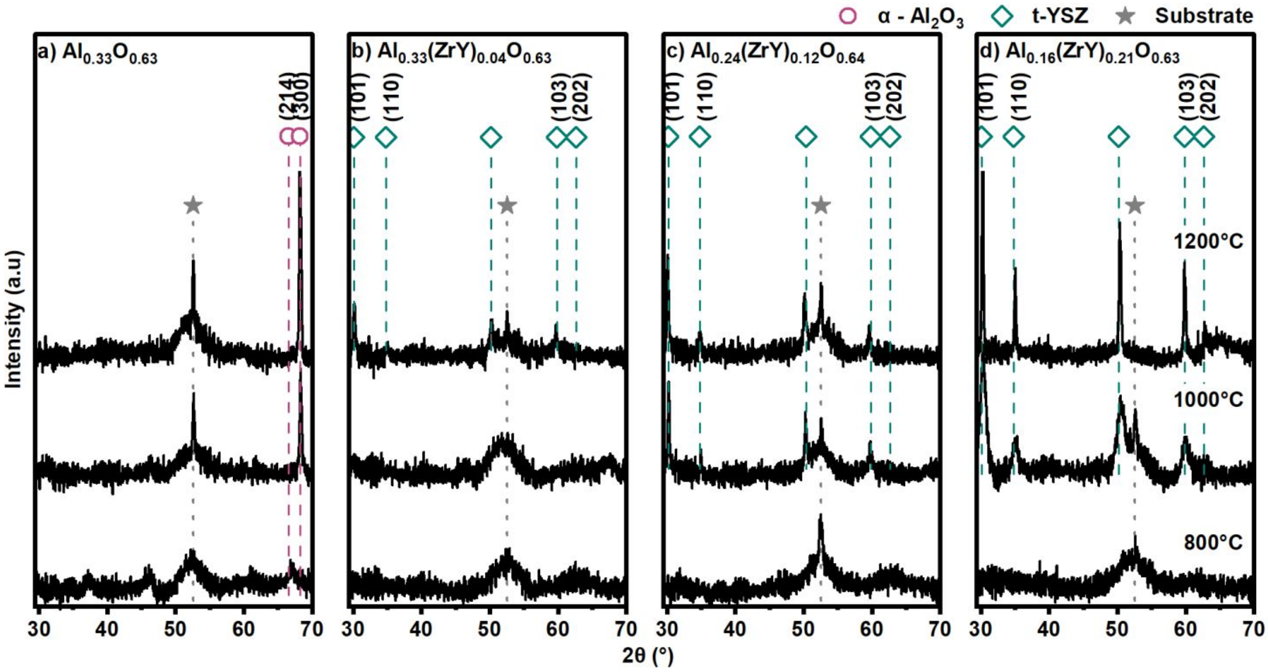

Figure S2: XRD three omega tilt diffractogram of as deposited and annealed samples in ambient air from 800°C to 1200°C in 200°C temperature steps. a) $Al_{0.37}O_{0.63}$, (b) $Al_{0.33}(ZrY)_{0.04}O_{0.63}$, (c) $Al_{0.24}(ZrY)_{0.12}O_{0.64}$ and (d) $Al_{0.16}(ZrY)_{0.21}O_{0.63}$.

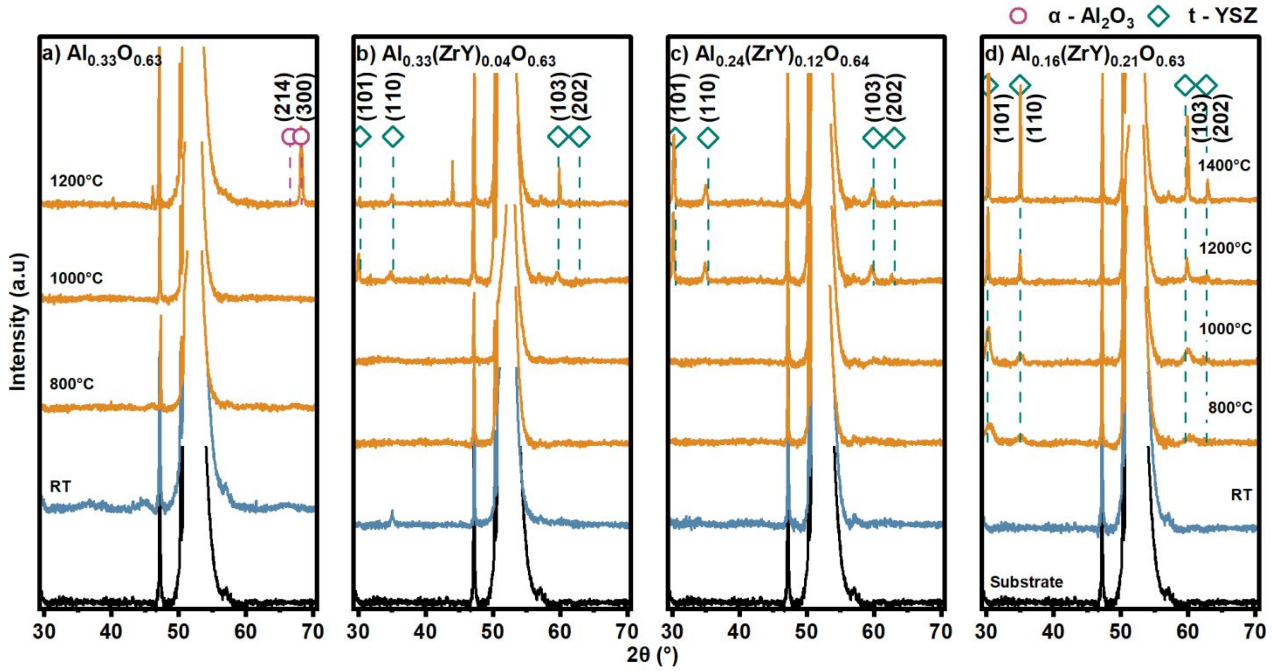

Figure S3: XRD diffractogram of as deposited and annealed samples in vacuum from 800°C to 1400°C in 200°C temperature steps. Substrate diffractogram is represented on the bottom as a reference. (a) $Al_{0.37}O_{0.63}$, (b) $Al_{0.33}(ZrY)_{0.04}O_{0.63}$, (c) $Al_{0.24}(ZrY)_{0.12}O_{0.64}$ and (d) $Al_{0.16}(ZrY)_{0.21}O_{0.63}$.

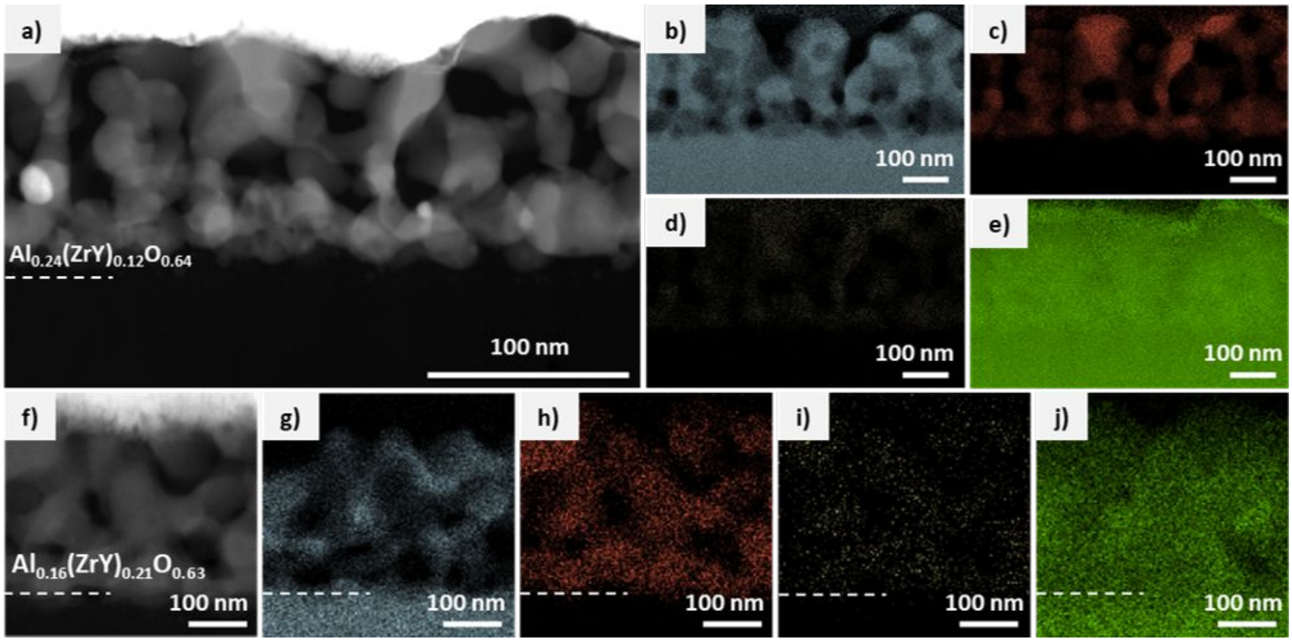

Figure S4: (a) STEM overview of $Al_{0.24}(ZrY)_{0.12}O_{0.64}$. EELS elemental mapping of (a) with the distribution of Al in (b), Zr in (c), Y in (d), and O in (e). (f) STEM overview of $Al_{0.16}(ZrY)_{0.21}O_{0.63}$. EELS elemental mapping of (f) with the distribution of Al in (g), Zr in (h), Y in (i), and O in (j).

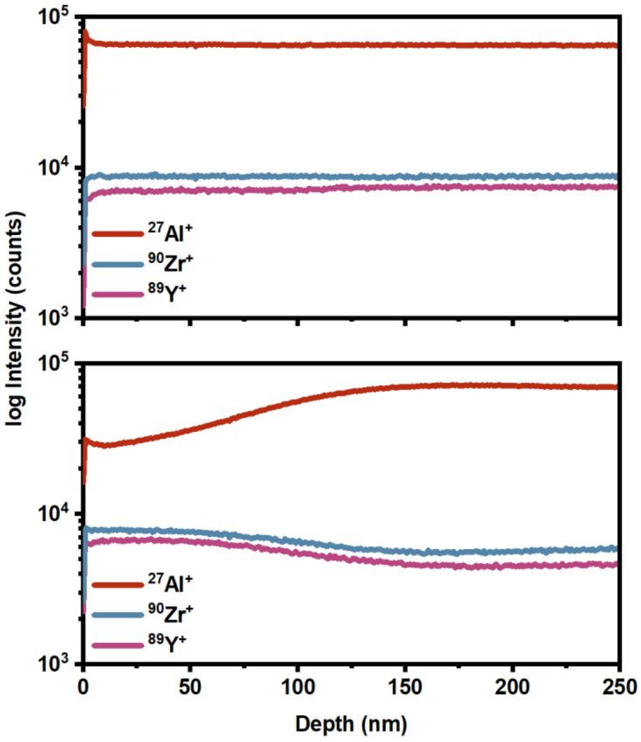

Figure S5: SIMS depth profile of $Al_{0.16}(ZrY)_{0.21}O_{0.63}$. (a) As deposited state, (b) Annealed samples at 1200°C.

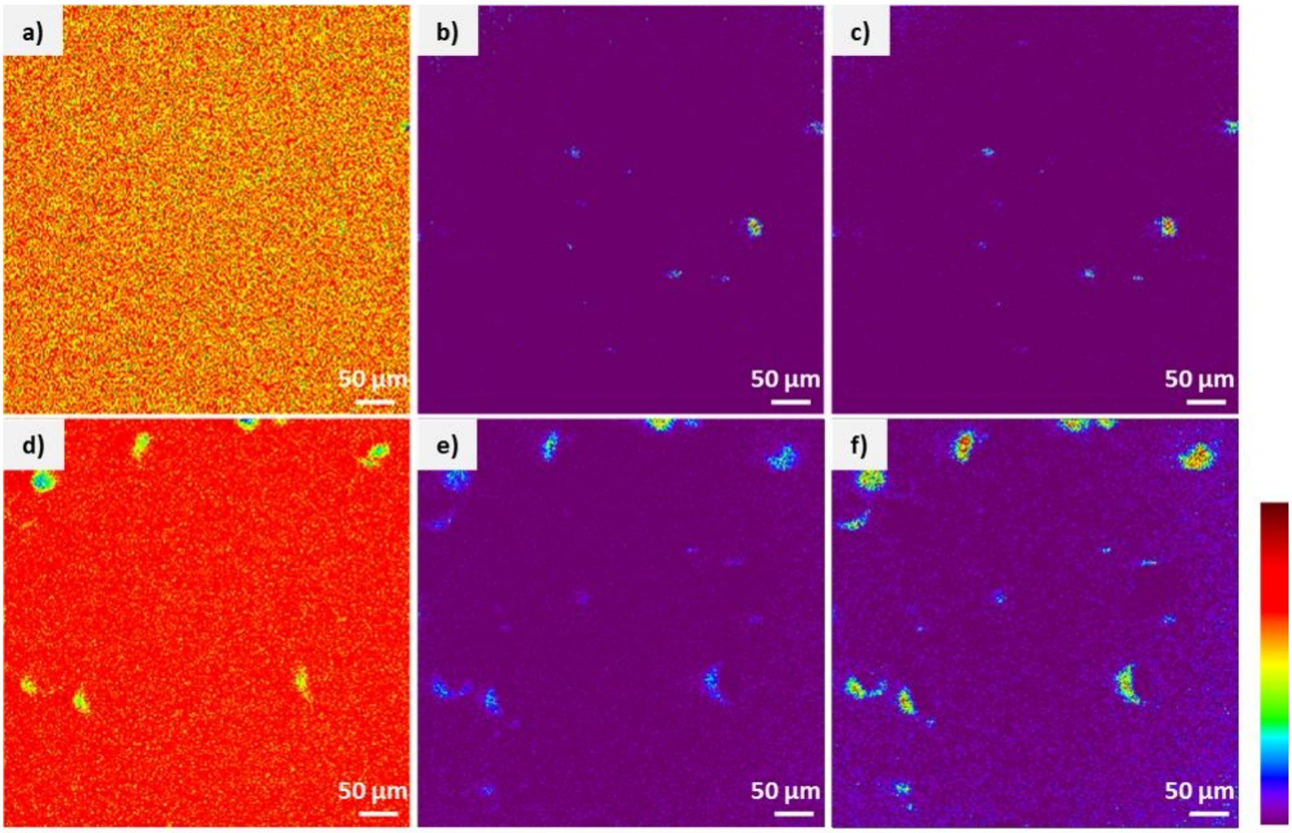

Figure S6: ToF-SIMS elemental mapping of the $Al_{0.16}(ZrY)_{0.21}O_{0.63}$. a) $^{27}Al^{+}$, b) $^{90}Zr^{+}$, c) $^{89}Y^{+}$ of the as deposited state. d) $^{27}Al^{+}$, e) $^{90}Zr^{+}$, f) $^{89}Y^{+}$ of the annealed state at 1200°C.

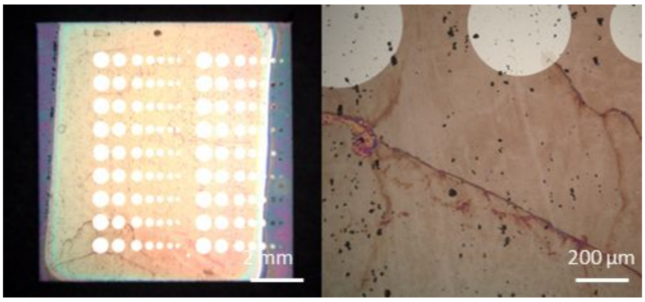

Figure S7: Light microscope image of the $Al_{0.37}O_{0.63}$ sample after thermal cycling during impedance measurements.

Temperature (°C)	$Al_{0.37}O_{0.63}$ ρ(Ω·m)	$Al_{0.33}(ZrY)_{0.04}O_{0.63}$ ρ(Ω·m)	$Al_{0.24}(ZrY)_{0.12}O_{0.64}$ ρ(Ω·m)	$Al_{0.16}(ZrY)_{0.21}O_{0.63}$ ρ(Ω·m)
300	$(2.285 \pm 0.005) \times 10^7$	$(5.2 \pm 1.8) \times 10^{10}$	$(6.35 \pm 0.03) \times 10^9$	$(6.7033 \pm 0.0002) \times 10^7$
325	$(1.376 \pm 0.003) \times 10^7$	$(2.9 \pm 0.5) \times 10^{10}$	$(2.96 \pm 0.01) \times 10^9$	$(3.9302 \pm 0.0001) \times 10^7$
350	$(8.51 \pm 0.02) \times 10^6$	$(1.4 \pm 0.1) \times 10^{10}$	$(1.990 \pm 0.008) \times 10^9$	$(2.3992 \pm 0.0001) \times 10^7$
375	$(5.30 \pm 0.01) \times 10^6$	$(7.30 \pm 0.04) \times 10^9$	$(1.090 \pm 0.004) \times 10^9$	$(1.5106 \pm 0.00006) \times 10^7$
400	$(3.364 \pm 0.007) \times 10^6$	$(3.55 \pm 0.02) \times 10^9$	$(2.7 \pm 0.9) \times 10^8$	$(9.7992 \pm 0.0007) \times 10^6$
425	$(2.189 \pm 0.004) \times 10^6$	$(1.73 \pm 0.06) \times 10^9$	$(1.7 \pm 0.9) \times 10^8$	$(6.4429 \pm 0.0008) \times 10^6$
450	$(1.416 \pm 0.003) \times 10^6$	$(8.40 \pm 0.02) \times 10^8$	$(9.4 \pm 0.3) \times 10^7$	$(4.1680 \pm 0.0005) \times 10^6$
475	$(9.51 \pm 0.02) \times 10^5$	$(3.98 \pm 0.09) \times 10^8$	$(4.9 \pm 0.1) \times 10^7$	$(2.6771 \pm 0.0002) \times 10^6$
500	$(6.59 \pm 0.01) \times 10^5$	$(1.84 \pm 0.04) \times 10^8$	$(2.7 \pm 0.3) \times 10^7$	$(1.7587 \pm 0.0001) \times 10^6$
525	$(4.748 \pm 0.009) \times 10^5$	$(8.60 \pm 0.1) \times 10^7$	$(1.4 \pm 0.2) \times 10^7$	$(1.1719 \pm 0.0001) \times 10^6$
550	$(3.578 \pm 0.007) \times 10^5$	$(4.18 \pm 0.06) \times 10^7$	$(7.64 \pm 0.08) \times 10^6$	$(7.6760 \pm 0.0006) \times 10^5$
575	$(2.787 \pm 0.005) \times 10^5$	$(2.11 \pm 0.02) \times 10^7$	$(4.45 \pm 0.05) \times 10^6$	$(4.6628 \pm 0.0004) \times 10^5$
600	$(2.235 \pm 0.005) \times 10^5$	$(1.07 \pm 0.01) \times 10^7$	$(2.55 \pm 0.02) \times 10^6$	$(3.2441 \pm 0.0004) \times 10^5$
625	$(1.844 \pm 0.004) \times 10^5$	$(5.70 \pm 0.01) \times 10^6$	$(1.52 \pm 0.01) \times 10^6$	$(2.3157 \pm 0.0003) \times 10^5$
650	$(1.544 \pm 0.003) \times 10^5$	$(3.188 \pm 0.006) \times 10^6$	$(9.439 \pm 0.008) \times 10^5$	$(1.7555 \pm 0.0002) \times 10^5$
675	$(1.317 \pm 0.003) \times 10^5$	$(1.829 \pm 0.003) \times 10^6$	$(5.767 \pm 0.005) \times 10^5$	$(8.8759 \pm 0.002) \times 10^4$
700	$(1.133 \pm 0.003) \times 10^5$	$(1.062 \pm 0.002) \times 10^6$	$(3.728 \pm 0.003) \times 10^5$	$(6.4579 \pm 0.0004) \times 10^4$
725	$(9.59 \pm 0.03) \times 10^4$	$(6.276 \pm 0.009) \times 10^5$	$(2.205 \pm 0.003) \times 10^5$	$(7.6293 \pm 0.0006) \times 10^4$
750	$(8.32 \pm 0.04) \times 10^4$	$(3.332 \pm 0.005) \times 10^5$	$(1.224 \pm 0.003) \times 10^5$	$(4.0984 \pm 0.0006) \times 10^4$

Table S1: Electrical resistivity values (in Ω·m) over temperature recorded in the cooling down cycle for $Al_{0.37}O_{0.63}$, $Al_{0.33}(ZrY)_{0.04}O_{0.63}$, $Al_{0.24}(ZrY)_{0.12}O_{0.64}$, and $Al_{0.16}(ZrY)_{0.21}O_{0.63}$.
